# Wearable Pulse Oximeter for Swimming Pool Safety

**DOI:** 10.3390/s22103823

**Published:** 2022-05-18

**Authors:** Elżbieta Kałamajska, Jacek Misiurewicz, Jerzy Weremczuk

**Affiliations:** Warsaw University of Technology, Institute of Electronic Systems, Nowowiejska 15/19, 00-665 Warsaw, Poland; elzbieta.kalamajska.stud@pw.edu.pl (E.K.); jacek.misiurewicz@pw.edu.pl (J.M.)

**Keywords:** swimming pool safety, pulse oximetry, biosensors

## Abstract

The purpose of this research was to develop an algorithm for a wearable device that would prevent people from drowning in swimming pools. The device should detect pre-drowning symptoms and alert the rescue staff. The proposed detection method is based on analyzing real-time data collected from a set of sensors, including a pulse oximeter. The pulse oximetry technique is used for measuring the heart rate and oxygen saturation in the subject’s blood. It is an optical method; subsequently, the measurements obtained this way are highly sensitive to interference from the subject’s motion. To eliminate noise caused by the subject’s movement, accelerometer data were used in the system. If the acceleration sensor does not detect movement, a biosensor is activated, and an analysis of selected physiological parameters is performed. Such a setup of the algorithm allows the device to distinguish situations in which the person rests and does not move from situations in which the examined person has lost consciousness and has begun to drown.

## 1. Introduction

An increasing interest in water sports and the construction of many new swimming pools raises the problem of pool safety. Thus, there is a need to create and develop safety surveillance systems for swimming pools [1]. Currently, such systems are scarce and have very limited detection ability.

One type of the existing pool safety systems is based on underwater video surveillance [2,3,4,5]. Refs. [6,7] propose systems with cameras installed above the water surface and with dedicated algorithms for the extraction of static and dynamic features. In [3], the prototype of a monitoring device built with a Raspberry Pi and a Pixy camera is described. Another interesting solution presents a system for large, open-water beaches that monitors using drones [8]. These systems, however, need good visibility conditions and may be unable to recognize drowning with many people in a recreational pool area or in a thermal water pool. In these cases, another type of system can come into the scene, namely, systems based on wearable devices with MEMS accelerometers and pressure sensors that are able to alert the lifeguard on duty [9]. Existing systems of this type usually trigger an alarm when a person stays long enough below a certain depth. Some systems also detect an atypical descent rate.

Most swimming pool accidents can indeed be modeled from data collected with a pressure sensor and an accelerometer; however, there may be dangerous situations that pass undetected (e.g., getting stuck at a depth that is too shallow to trigger the alarm). In order to prevent drowning in such unusual cases, an optical heart rate and oxygen saturation sensor—a pulse oximeter—could be added to the sensor wristband.

Pulse oximeters make it possible to detect a drop in oxygen saturation, which may indicate the loss of consciousness under water and the initial stage of drowning. The addition of a small optical sensor to commonly used pool wristbands is a convenient solution both for swimming pool visitors and staff. However, no such devices available on the market can be treated as a “gold standard”. A wearable anti-drowning wristband was introduced in [10], wherein the authors proposed a system with a pulse oximeter, a pressure sensor, and an accelerometer, and combined it with a simple threshold algorithm. However, threshold algorithms can malfunction because each human has a different biological background. Therefore, it is necessary to adjust those parameters individually. In the present paper, we describe an algorithm that allows us to detect pre-drowning symptoms without relying on predefined thresholds for individual vital signs.

The proposed sensor system aims to improve the probability of drowning risk detection when keeping the false alarm rate sufficiently low. The improvement comes from analyzing vital signs and reacting to their deterioration. The work presents a combination of motion artifact-resistant algorithms, along with its monotonicity analysis. The algorithms are based on digital filtering and fast Fourier transform.

### Five Stages of Drowning

When it comes to drowning detection, it is helpful to know how the classic case of drowning proceeds and how the examined parameters (heart rate and blood oxygen saturation) change during its course. Drowning can usually be described in five phases [11].

The first phase lasts for a few seconds, as the person tries to catch a breath. During the second phase, the person holds his/her breath to prevent water from getting into the lungs. Up to this moment, the subject is conscious and usually makes chaotic movements, making it challenging to measure physiological parameters. Blood oxygen saturation decreases because the oxygen supply is cut off, while the heart rate accelerates to provide the right amount of oxygen to the body.

The third phase begins when a person stops breathing and loses consciousness. The developed algorithm detects drowning precisely in this phase since the person is stationary and optical measurement of parameters is possible. It is also the last moment to save someone before risking serious health consequences associated with brain hypoxia. This stage lasts up to a maximum of 60 s, a period during which both oxygen saturation and heart rate decrease.

Phase four and five are the stages in which hypoxia of the brain causes irreversible effects and then death. It is necessary to detect drowning not later than in the third phase in order to prevent this from happening.

## 2. Materials and Methods

**Figure 1:**
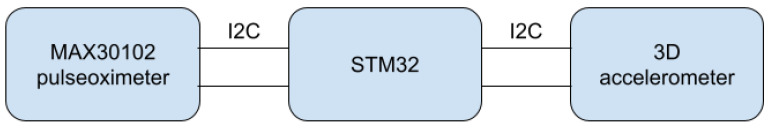
Main components of the system.

The proposed method was implemented and tested in a wristband built around an ARM low-energy microcontroller from the STM32 family. The algorithm is based on continuous data processing from two sensors: the accelerometer (low cost, MEMS type, ±6 g range, 0.5% accuracy and non-linearity, respectively) and the pulse oximeter (self-made, using OEM wrist band optics delivered by the Berserg company). On their basis, it is possible to classify what happens to a person in the swimming pool area. The main components of the system are presented in Figure 1.

The pulse oximeter serves two purposes. One function, always active, is to check if the measuring device is still on the wrist so that accidentally dropping the band into the water does not trigger an alarm. The second function is to monitor vital parameters, namely, pulse and oxygen saturation. These parameters should be measured while a person remains motionless or if any occurring hand movements are slow and light. Otherwise, the noise corresponding to the motion artifact can be many times greater than the component of the signal associated with heart rate.

The use of an acceleration sensor allows us to determine whether the data from the pulse sensor will be valid and useful. In the case of movement detection, the system can turn off the pulse oximeter for better energy management, which is crucial in a small wearable device.

**Figure 2:**
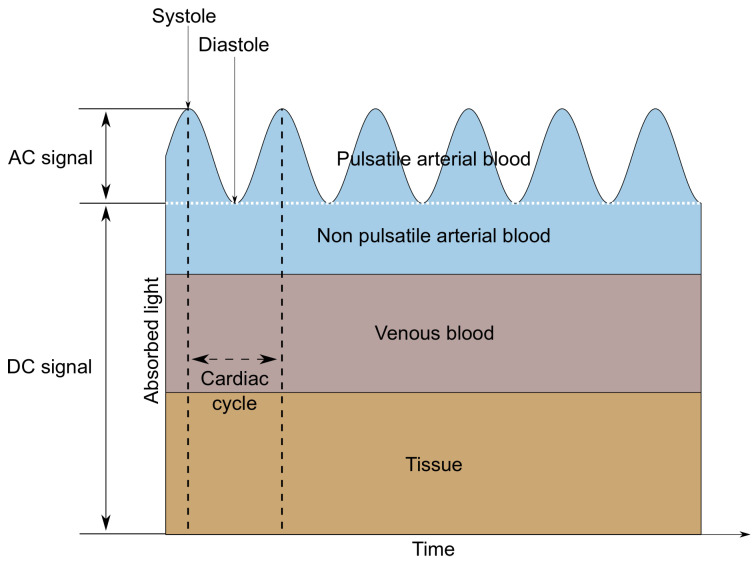
AC and DC components of a PPG signal.

A MAX30102 LED-based reflective-type heart rate and oxygen saturation sensor was used in the tested wristband. This sensor was chosen due to the built-in photodiode and the red and infrared diodes recommended for oxygen saturation measurements [12,13]. MAX30102 is a low-noise optical sensor and it provides ambient light rejection in the processing channel [14]. Applying the sensor to the skin, with one diode turned on, allows us to observe changes in the intensity of the waves reflected from the tissues using a photodetector. The shape of the reflected signal (called PPG—Photopletysmography signal) is related to the volumetric changes of the arteries and capillaries. It is the characteristic ripple that occurs at regular intervals, which is presented in Figure 2. Only the AC component is needed to determine the heart rate, so it is possible to measure this parameter using only one diode. When alternately illuminating the skin with light sources of different wavelengths—in this case, red (660 nm) and infrared (880 nm)—it is also possible to determine the value of oxygen saturation, which describes how much hemoglobin is currently bound to oxygen, compared to the total hemoglobin (oxygenated and deoxygenated) content in blood [15]. This technique is based on measuring the difference in the absorption or reflection coefficients of oxygenated and deoxygenated hemoglobin for different wavelengths.

The research aimed to determine the heart rate and oxygen saturation values in the best possible way and to make a reliable decision on the eventual danger based on the determined values. The significant factors in the selection of the measurement methods were precision, low computational complexity, and short delay. Another critical factor in a safety system is a low false-positive rate—the occurrence of frequent false alarms may make the rescue staff bored and may lead to ignoring the true alarm.

We used data collected using the authors as test subjects in the presented research. All measurements taken within this article were performed during controlled breath holding. For obvious reasons, we did not want to enter the unconscious phase.

## 3. Heart Rate Algorithm

Raw MAX30102 data—the photoplethysmography signal—consists of AC and DC components. The DC component corresponds to non-pulsatile tissue, while the AC component alternates according to the heart cycle. Only the variable part of the signal is relevant for heart rate determination; thus, the mean value is usually subtracted from the signal used in the HR (heart rate) measurement.

The HR measurement may be based on determining the time between consecutive systolic peaks or on the measurement of the primary frequency component of the signal. Articles [16,17] describe the algorithm for HR determination with the use of the MUSIC (multiple signal classification) technique. This problem was also approached with the use of the wavelet transform in work [18]. In our setup, a fast Fourier transform (FFT) algorithm was used to determine the heartbeat frequency, as it is well optimized and has low computational complexity.

**Figure 3:**
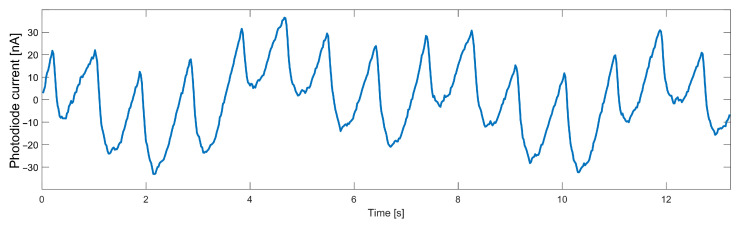
Data measured from an optical heart rate sensor.

The AC component of data collected from the heart rate sensor, even when no movement was detected, is characterized by a noise that significantly worsens the FFT result. For a particular frequency spectrum to be useful, it was necessary first to remove high-frequency noise from the signal. Another significant disturbance is breathing, the frequency of which is close to the frequency of the useful signal. The necessity to remove such a component forces the use of a higher-order filter, which will have an appropriately steep transition characteristic. The variable component of the signal measured on a finger with the infrared diode turned on is shown in Figure 3. The breathing component is clearly visible in a slower sinusoid.

A bandpass filter has been designed to filter out the interfering components properly. It was important that the filter had the flattest possible passband characteristics and a not-too-high order, as the filter order directly affects the delay and the computational complexity of the algorithm. However, lowering the order of the bandpass filter leads to a significant deterioration of its amplitude characteristics—the bandwidth narrows, and bandpass edges become more rounded.

**Figure 4:**
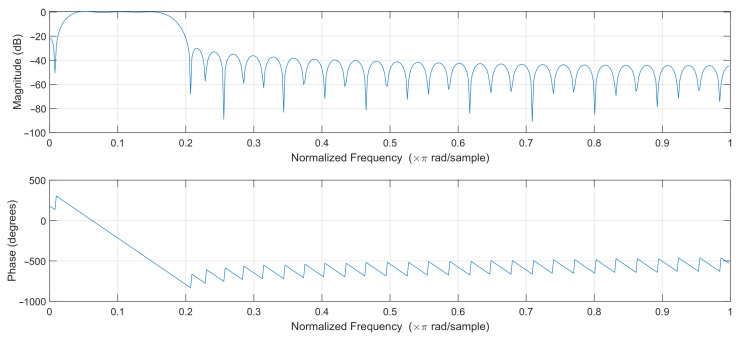
Filter frequency response.

In order to select the optimal filter, least-squares linear-phase filters of different orders were compared, and a filter of order 64 was selected. The filters with lower orders gave unsatisfactory results, while higher-order filters introduced a slight improvement at the cost of additional computational effort. The passband of the designed filter was selected to effectively pass frequencies corresponding to the pulse rate in the range of 45–250 bpm. The magnitude and phase response of the designed filter are shown in Figure 4. The filter works at 50 samples per second, so the normalized frequency of 0.2 corresponds to 5 Hz or 300 bpm (beats per minute).

**Figure 5:**
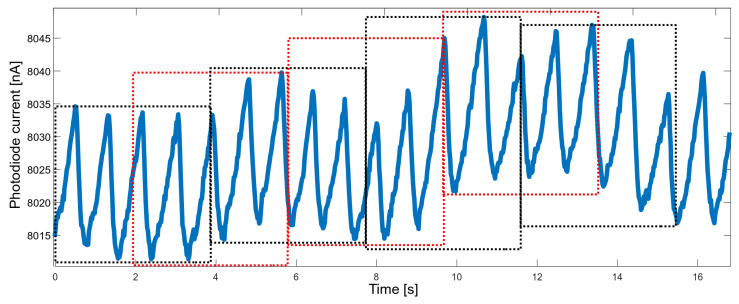
Raw PPG data divided into overlapping time windows (red and black boxes).

Another property that needed to be balanced was the response time. When measuring vital signs in order to raise the alarm, the response should be fast enough; on the other hand, the accuracy of the measured parameters should not suffer too much. It was decided to determine the values of the parameters based on approximately four-second segments. A four-second time window allows gathering, on average—for a heart rate of 60 bpm—four peaks, which allows for a reliable determination of the heart rate. To improve performance, segments overlapping at 50% were used (Figure 5).

The sampling rate of the heart rate sensor was set to 50 Hz. Without performing additional operations, this allowed us to determine the discrete Fourier transform (DFT) spectrum with a frequency range up to 25 Hz and relatively poor spectral resolution. When determining the heart rate value, the lower frequencies, up to about 4 Hz, are significant, while the higher ones are unnecessary. In order to avoid performing redundant calculations, it was decided to apply decimation by a factor of 2. This reduces the spectrum’s frequency range and allows for lowering the filter’s computational complexity twice.

The proportional reduction in the number of samples *N* and the sampling rate $f_{s}$ does not affect the spectral resolution Δf. Finer spectral sampling was obtained by increasing the signal length with the zero-padding technique. In this technique, zero values are appended to the original signal before performing DFT calculations. Those additional samples do not worsen the frequency spectrum. Instead, they allow us to estimate the heart rate frequency more accurately [19]. After padding the data with zero samples to the length of 512, the frequency resolution was improved by a factor of approximately 4. This was a consequence of the following Formula (1) [19]:

(1) $$\Delta f=\frac{f_{s}}{N}$$

Performing FFT on zero-padded data is equivalent to enforcing a rectangular window over the dataset. The use of a rectangular window is associated with several disadvantages, for example, spectral leakage. This effect causes the appearance of additional side lobes in the frequency spectrum. It is possible to reduce the spectral leakage and lower the side lobes’ level by applying different windows. Windowing is achieved by multiplying the time-domain data by a chosen window function [19,20]. The Hamming window, a symmetric, bell-shaped curve, was used to reduce the mentioned effects. The length of this window is equal to the length of the original signal after downsampling.

**Figure 6:**
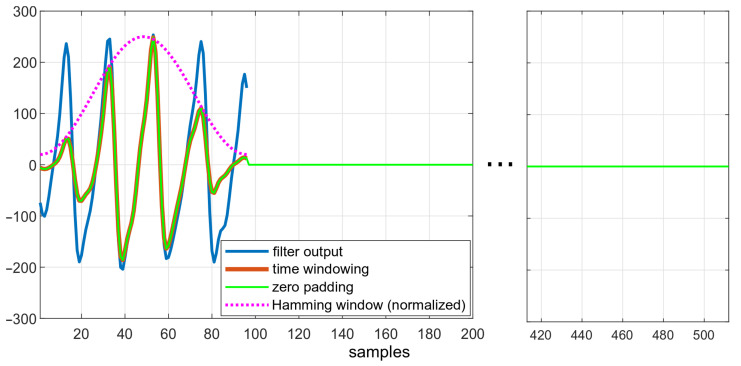
Data in different states of the algorithm: comparison of filtered data, filtered data after applying the Hamming window, and zero-padded data.

Figure 6 depicts the effects of subsequent steps of the algorithm. The downsampled data, smoothed after filtration, are shown in blue. The effect of enforcing the Hamming window is visible in red—amplitudes of ripples both at the beginning and at the end of windowed dataset is lower than in the original data.

**Figure 7:**
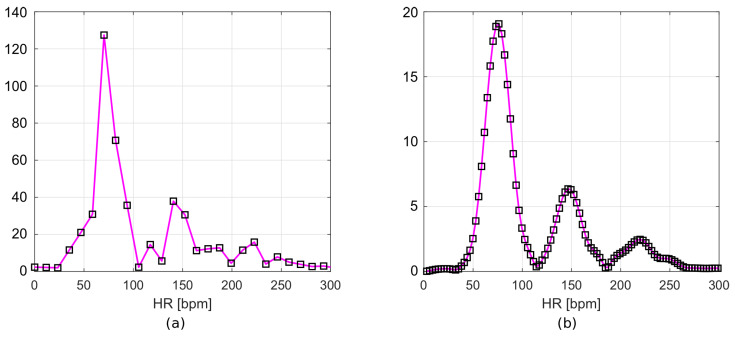
Frequency spectrum of (**a**) filtered data and (**b**) filtered data with time-windowing and zero-padding.

The resulting frequency responses are shown in Figure 7a,b. Figure 7a shows the frequency spectrum of a signal after filtration, but without zero padding. Figure 7b includes the Hamming window and zero values appended to the signal. The frequency resolution of the first plot (Figure 7a), according to Equation (1), is 0.195 Hz. This corresponds to 11.72 bpm, while for the second plot (Figure 7b), it is 0.049 Hz (2.93 bpm).

**Figure 8:**
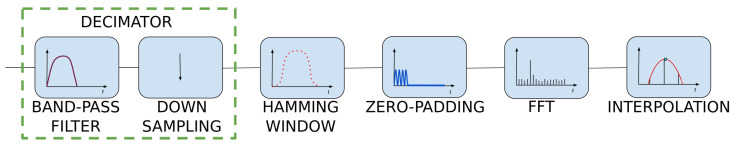
HR measurement block diagram.

Heart rate can be determined from the location of the maximum in the calculated spectrum. For a more precise determination of the HR value, the spectral samples can be interpolated—we use a polynomial of the second degree here. Thus, the maximum of the interpolated spectrum is determined on the basis of three samples: the maximal one and two adjacent ones. Finding this maximum requires solving the system of three equations: $f\left(x_{i}\right)=a{\left(x_{i}\right)}^{2}+b\left(x_{i}\right)+c$, finding the a, b, and c coefficients and the extreme $p=-\frac{b}{2a}$. The summary of the algorithm’s operation is presented in Figure 8.

## 4. Oxygen Saturation Algorithm

Oxygen must be constantly transported from the lungs to all organs for the body to function properly. For healthy people, oxygen saturation remains at the level of 95–100% [21]. This value can drop during exhaustive training, when the circulatory system does not keep pace with the oxygen supply, or when the oxygen is cut off. Cutting off the oxygen supply by being unconsciousness underwater for a few minutes may cause irreversible damage to the brain or even death. Monitoring this parameter makes it possible to react much earlier and avoid serious accidents.

In order to obtain the oxygen saturation value, the MAX30102 sensor alternately illuminates the body with red and infrared diodes. Both obtained signals should be processed in the same way as described in the HR algorithm. The mean values of the raw data (DCred and DCir) and the maxima of the frequency spectrum (ACred and ACir) allow the blood oxygen percentage to be determined according to Equation (2), supplied by the sensor manufacturer [22].

(2) $$SPO2=104-17\cdot \frac{\frac{AC_{red}}{DC_{red}}}{\frac{AC_{ir}}{DC_{ir}}}$$

## 5. Decision Algorithm

**Figure 9:**
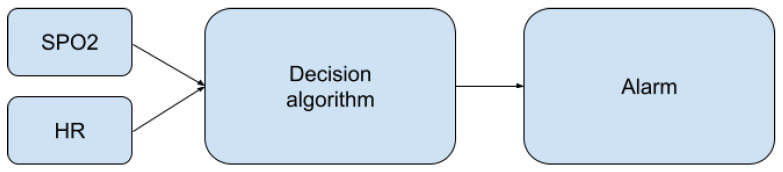
Place of the decision algorithm in the alarm-triggering process.

The presented algorithms enable the determination of heart rate and oxygen saturation values based on fragments of the PPG signal. At the stage of the decision algorithm, some of the resulting values are classified as disturbed and are rejected. The rest of them are used to decide about the condition of the person staying in the pool (Figure 9).

The developed decision algorithm aims to detect all situations in which the monitored person begins to lack oxygen. The single value of saturation does not allow for such a decision. Optical measurements are dependent on factors such as optical path length or reflectance. There will be differences for people with different skin tones or different amounts of muscle tissue [23]. For the measurements to be useful, instead of reacting to low oxygen saturation values, it is necessary to detect their decrease. This approach significantly reduces the number of false positives and allows for the effective detection of dangerous events and quick reactions.

To detect decreases in oxygen saturation, the measurements recorded for the previous 20 s were considered, giving—without any disturbances—10 observations. On their basis, the slope of the trend line was calculated by applying a simple linear regression model to valid data. The observed signals are very susceptible to movement disturbances. In order to avoid false alarms, outliers must be detected and removed before further data processing.

The decision algorithm consists of the following steps:

1. The rejection of disturbed HR values;
2. The rejection of disturbed oxygen saturation values;
3. Linear regression;
4. The analysis of heart rate and oxygen saturation regression slopes.

### 5.1. The Disturbed HR Values

The HR algorithm presented above works well with uncorrupted data but gives erroneous results in the case of slight hand movements or if the pressure on the sensor is not stable. Interferences occurring in the PPG signal due to hand movements are often impossible to remove with a classic digital filter, because their frequency is in the useful frequency range, and their amplitude is many times greater than the PPG ripple amplitude. Despite this, as a result of processing the disturbed PPG signal using the presented algorithm, the component corresponding to heart rate in the frequency spectrum is visible as one of the local maxima. If the measurement contains movement artifacts, the global maximum from the frequency spectrum may not be related to the heart rate but to the motion artifact. Therefore, the algorithm has been slightly improved, allowing for better HR determinations.

**Figure 10:**
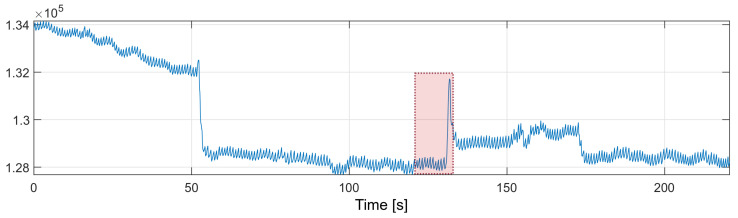
PPG signal with motion artifacts (red box marks the fragment discussed in the text).

In order to improve the performance of the algorithm, The nearest-peak selection method was used, which is a new method of removing MA disturbances. The method is based on the analysis of the disturbed PPG frequency spectrum. The improvement comes from the assumption that the global maximum of the frequency spectrum may not correspond to the heart rate component. All maximal local values are selected from the spectrum, and the most probable maximum is determined from among them, i.e., the maximum closest to the heart rate values determined in the previous time periods. The fragment marked in red in Figure 10 will be thoroughly analyzed.

**Figure 11:**
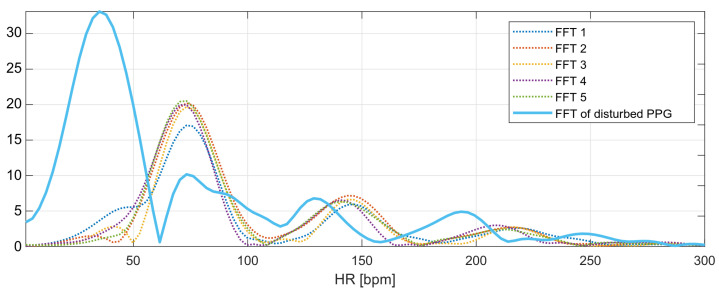
Frequency spectra of the analyzed PPG signals.

The marked fragment contains six consecutive four-second fragments of the PPG signal with a 50% overlap, the spectra of which are presented in Figure 11. The spectra of the first five signal fragments (labeled FFT1–FFT5) correspond to the undisturbed PPG signal. The heart rate values determined for the undisturbed fragments are presented in Table 1.

The heart rate values determined from the undisturbed fragments of the PPG signal are around 73.1 bpm (average from Table 1). This is the expected value for the next heart rate measurement. The last spectrum, the spectrum of the disturbed PPG signal, has a global maximum corresponding to the heart rate value of 35 bpm; however, this value is not related to the heartbeat, but to recorded motion artifacts.

The local maxima from the disturbed PPG signal spectrum correspond to the following values of the heart rate: 35.16, 73.24, 128.91, and 193.36 bpm. The nearest-peak selection method is based on finding the heart rate value closest to the expected value (73.1 bpm) among the local maxima; therefore, the new heart rate value will be taken as the frequency corresponding to the second local maximum of the spectrum: 73.24 bpm.

### 5.2. The Disturbed Oxygen Saturation Values

For oxygen saturation, some of the results were discarded due to large variations of the saturation values. The oxygen saturation values can be described by the normal distribution. One measure of variability is the interquartile range, IRQ, which describes the range of the middle half of the dataset, that is, the distance between the first quartile Q1 and the third quartile Q3 [24].

**Figure 12:**
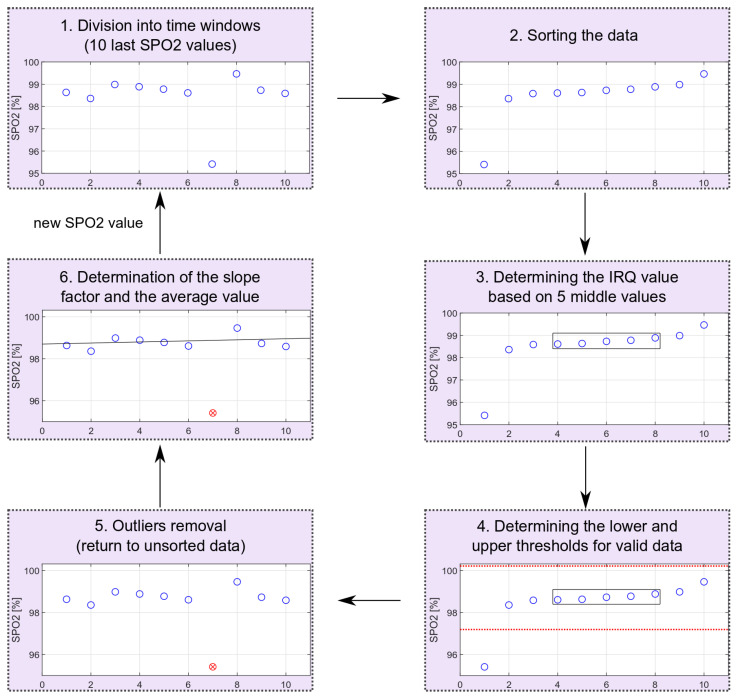
Outliers’ removal scheme (red circle marks an identified outlier).

The outliers’ removal is performed locally for consecutive time windows, including 10 measurements (20-s fragment of the PPG signal). The oxygen saturation values are sorted and based on the middle values from the sorted sequence; the IRQ (Equation (3)) and the lower (Equation (4)) and upper (Equation (5)) limits for non-outliers’ data are determined. After receiving the next oxygen saturation value, all values rejected in the previous calculations are reconsidered. The scheme of removing extreme values is shown in Figure 12. The schema shows the entire process for data with one outlier. The proposed method allows for dynamic threshold settings for valid data.

(3) $$IRQ=Q_{3}-Q_{1}$$

(4) $$SPO2{}_{lowertrsh}=Q_{1}-10\cdot IRQ$$

(5) $$SPO2{}_{uppertrsh}=Q_{3}+10\cdot IRQ$$

### 5.3. Truncated Linear Regression

To describe the relationship between successive values of saturation, a linear regression was used, applied only for selected data (data without outliers), i.e., a truncated linear regression. The oxygen saturation slope estimator $\hat{b}$ is determined using the ordinary least squares method, OLS. It is described in the following formula (Equation (6)) [25].

(6) $$\hat{b}=\frac{cov(t,SPO2)}{var(t)}$$

where:

SPO2 is a vector of valid oxygen saturation values without outliers;
*t* is a time vector.

### 5.4. The Analysis of Heart Rate and Oxygen Saturation Regression Slopes

The alarm should be triggered as a result of a prolonged decrease of heart rate and oxygen saturation. The alarm triggered based on a single regression slope with a value below a certain threshold appeared far too often, due to the oxygen saturation fluctuations. The nature of HR and SPO2 changes that should trigger an alarm is described by the following threshold values:

- SPO2 slope threshold;
- HR slope threshold;
- time threshold.

The SPO2 slope threshold indicates the lowest oxygen saturation slope value that is not considered dangerous. This threshold has been set at −0.05 $\frac{\%}{s}$. The same slope threshold for the heart rate value was set at −0.2 bps. Exceeding those two values increases the time_counter, which overflows when the time threshold set for five consecutive measurements is reached.

The SPO2 slope threshold has been determined based on dry measurements with breath holding (an example with a measurement of this type is provided in the Results Section 6.3). Unfortunately, it was impossible to test the HR slope threshold, because during the breath-holding measurements, the HR rose. The fall of the HR value appears only after consciousness is lost. For the detection of drowning, the time threshold can be increased.

The time threshold has been set at a level that allows the algorithm to be tested safely and triggered by holding the breath. The experiments showed that, during the early stage of breath holding, there was no visible change in the level of SPO2 (for a tested subject, it was approximately 40 s). However, this delay of 40 s should only occur during breath-holding measurements. After loss of consciousness, the decrease in SPO2 should be visible immediately, because in this phase of drowning, oxygen has already been cut off for some time. The alarm is triggered when a decrease in HR and SPO2 values is detected. The slope is determined based on 10 measurements, i.e., 20 s, and must be maintained for 5 (time threshold) consecutive measurements. Each next slope is recalculated with a new SPO2 measurement (every 2 s). Thus, the alarm is triggered, at the latest, 30 s after the beginning of the decrease in these values.

**Figure 13:**
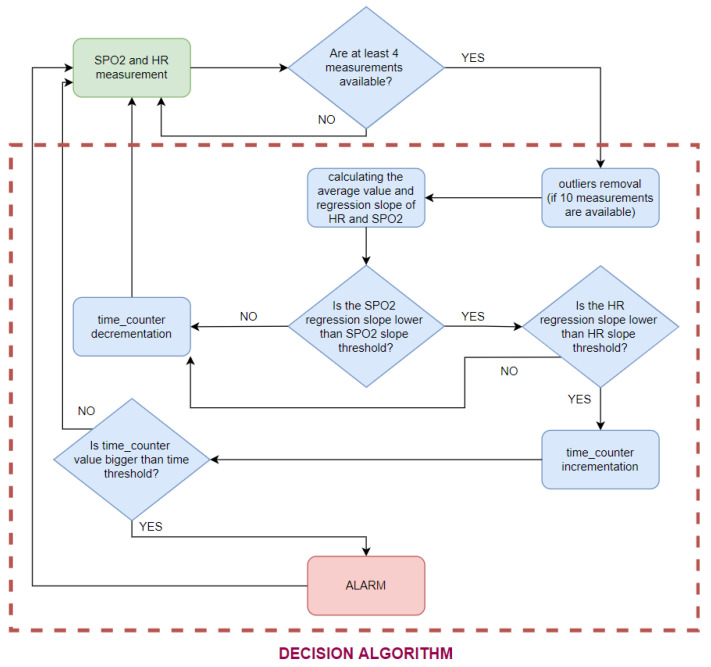
Decision algorithm scheme.

The diagram of the decision algorithm operation is shown in Figure 13. To shorten the delay at the beginning of the measurement (when a person stops moving), the authors decided to calculate the slope on the basis of at least four SPO2 and HR values. If less than 10 measurements were available, no outliers were removed.

## 6. Results

We performed a set of preliminary tests using the authors as test subjects and with the purpose of verifying the concept. More extensive tests are planned in further steps of the device’s development. The algorithm tests were performed in MATLAB, and the final implementation was made for the STM32 microcontroller. The tests presented below focus on the variance of the tested parameters and noise immunity. The decision algorithm (including only oxygen saturation values) has also been tested during the rest period, and while holding the breath, to simulate drowning.

### 6.1. Heart Rate Noise-Cancellation Algorithm Testing

**Figure 14:**
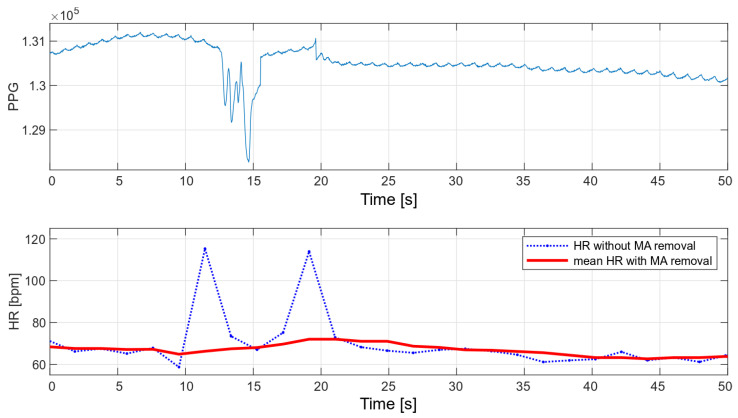
HR values for a disturbed PPG signal.

**Figure 15:**
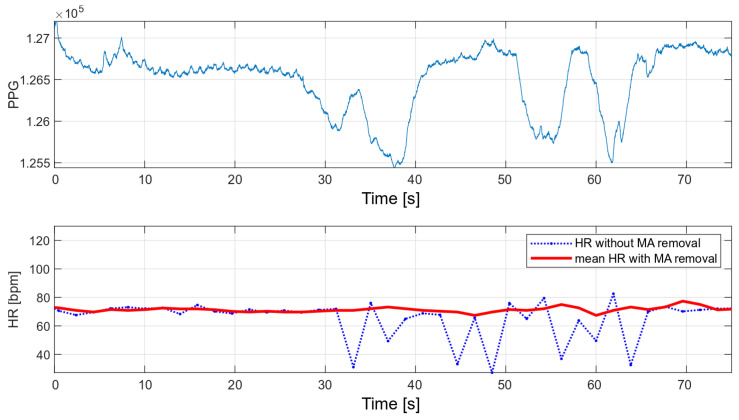
HR values for a disturbed PPG signal.

The main advantage of the developed algorithm is its high resistance to disturbances during motion. As part of the interference immunity tests, multiple measurements were made during small hand movements and with changes in pressure on the optical heart rate and oxygen saturation sensor. Any slight disturbance significantly affects the signal measured with the oximeter. Figure 14 and Figure 15 show the raw PPG signal with visible noise and its corresponding heart rate values. Two heart rate values have been summarized in the charts: raw heart rate and heart rate moving average, with disturbed values’ removal (nearest-peak selection method).

The applied method of removing disturbances significantly improves the final heart rate values. A comparison of the variance for the two collected signals before and after the removal of motion artifacts is shown in Table 2.

### 6.2. Dependence of Oxygen Saturation Deviations from the Place of Measurement—Comparison of the Measurements on the Finger and the Wrist

Medical oxygen saturation measurement devices are characterized by high accuracy, which is possible, among other things, due to their design. These are usually finger measurements in which the oximeter is attached with a special clip that provides a stable pressure. Wrist measurements present a greater engineering challenge, as they are associated with higher noise levels and more distortion during movement. In addition, the finger is the area with more blood supply, so the measured variable component is much stronger than during wrist measurements.

**Figure 16:**
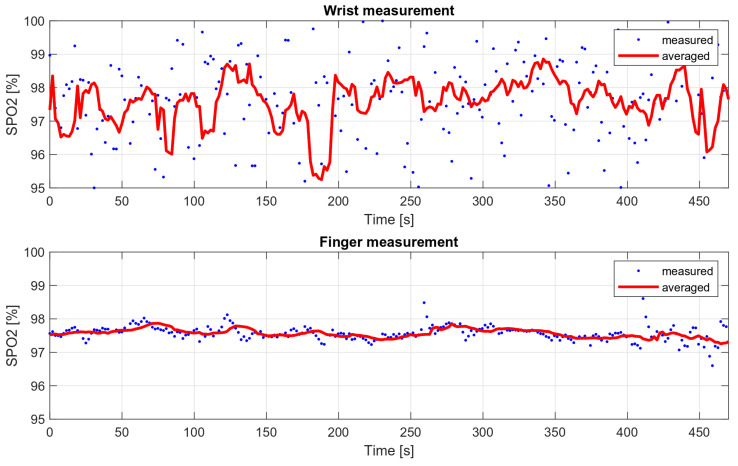
Wrist and finger oxygen saturation measurements’ comparison.

In order to compare the deviations of saturation for the wrist measurement and the measurement on the finger, two eight-minute measurements were taken. The wrist measurement was conducted with the wristband fastened loosely, to show how it affects the results. The SPO2 values obtained during these measurements are shown in Figure 16, and their variances are summarized in Table 3.

**Figure 17:**
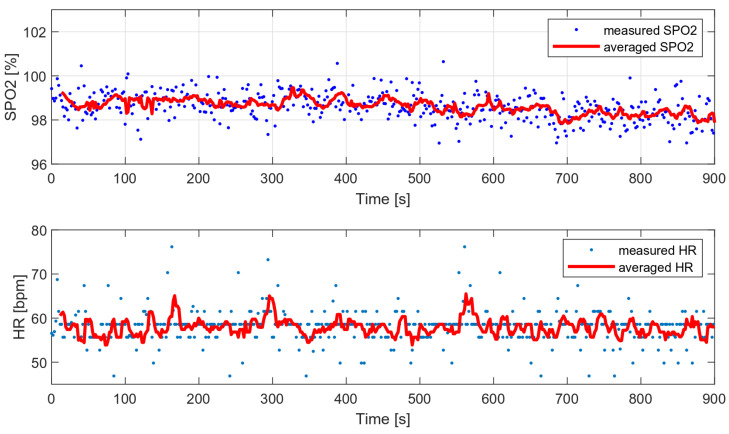
HR and SPO2 measured during sleep (data with low level of motion artifacts).

As part of the comparative tests, a 12-min HR and oxygen saturation wrist measurement was performed with a very low level of disturbance (data collected during sleep) and with the wristband fixed properly. Figure 17 presents the measured and average vital parameters collected during this measurement.

The variances of the obtained values were compared with the variances of the selected algorithms and are presented in Table 4. The following algorithms were analyzed:

- MUSIC (multiple signal classification) algorithm—only HR;
- Wavelet transform;
- Decimation filter + periodogram.

The variance of the tested algorithm for data without significant movement artifacts is comparable to the variance of those parameters determined on the basis of the periodogram and smaller than the variance obtained with the use of the wavelet transform.

**Figure 18:**
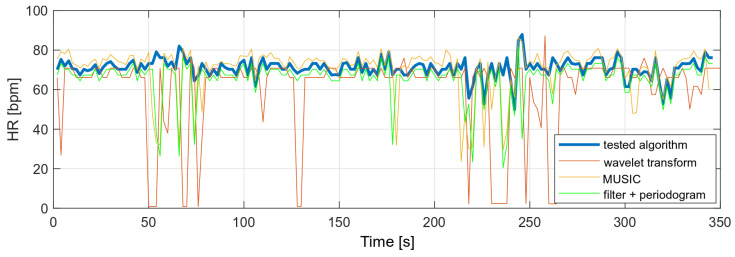
Comparison of HR values (data with high level of motion artifacts).

The same algorithms were used to compare heart rate values with a higher level of MA in the PPG signal. The results showed that the other algorithms would require some of the results to be rejected or measurements to be taken at a lower MA level. The tested algorithm, despite the disturbances, did not show any higher variances than in the case of the measurement during stillness. A comparison of the obtained heart rate values is shown in Figure 18.

### 6.3. Breath-Holding Tests

**Figure 19:**
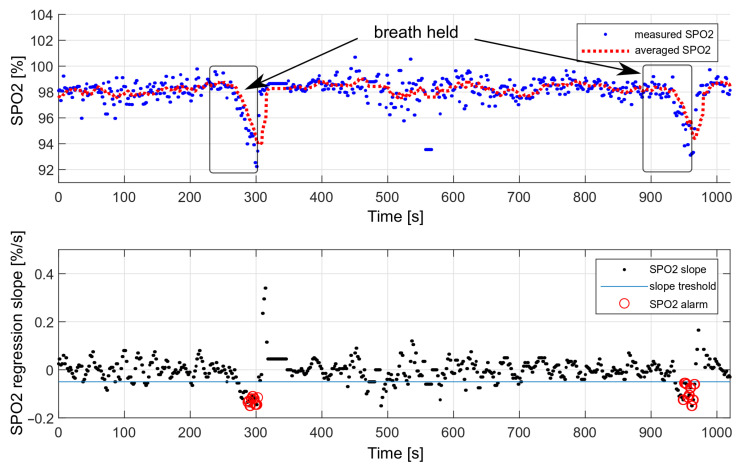
Testing the effectiveness of triggering an alarm with a decrease in oxygen saturation.

The performance of the decision algorithm was tested during longer breath-holds as part of a drowning simulation. The tests were performed only considering the oxygen saturation values. A simulation of drowning with both HR and SPO2 values was not possible, as it would require the actual loss of consciousness. During the presented measurement, the breath was held twice, each time for about 70 s. The effect is shown in Figure 19.

We performed 50 breath-holding tests in a dry environment. The drowning detection rate was estimated at 90%, based on the number of triggered alarms. Each measurement consisted of holding the breath for 70 s. Some measurements did not trigger the alarm because the oxygen saturation drop was too short and was not considered dangerous.

The false-alarm rate depends on the level of the motion artifacts. For multiple measurements carried out during sleep, no false alarms were recorded; however, during slight hand movements and with the band loosely fastened, false alarms occurred, on average, every 30 min.

## 7. Discussion

The algorithm described in this article is aimed at detecting a decrease in oxygen saturation and heart rate when a person loses consciousness and becomes still. In this case, the tests were carried out during controlled breath holding, and it was only possible to check the algorithm reacting to the decrease in oxygen saturation. The slope coefficient of the heart rate value should be determined after recording the actual cases of drowning.

The use of two thresholds, both for HR and SPO2, eliminates false drowning detections associated with, e.g., long breath holding under water. The situation when both heart rate and oxygen saturation decrease is unique and cannot be reproduced in a controlled way. Holding your breath under water causes a strong increase in heart rate, during which the alarm will not be triggered.

Breath-holding tests were performed outside of the swimming pool with the purpose of verifying the concept. The tests have shown that optical SPO2 measurements are feasible and give reliable results. Being aware of obvious limitations of the “dry” experiments, the authors plan more extensive tests of the developed algorithm, including underwater measurements, in further steps of the device’s development. Some interesting measurements are reported in [26]. It must be, however, noted that they were performed with a pulse oximeter placed on the finger, nostril, or earlobe, and that the actual measurements were taken on the beach—after the subjects had left the water.

The typical swimming pool disturbances were taken into account during the development of the algorithm. The algorithm is resilient to minor disturbances resulting, e.g., from water undulations or pressure changes due to the activity of other swimmers. These are usually low-frequency interferences that are removed with a digital filter. Single larger disturbances, e.g., due to hypoxic convulsions, would be removed by the nearest-peak selection method, or the measured SPO2 value would be classified as an outlier and discarded. There are also other methods mentioned in the literature to remove interference—for example, methods based on the Kalman filter [27].

For the optical measurement to work, the sensors must adhere to the skin in a stable way and should not move during the measurement. This necessitates wearing the wristbands in a tightly fixed manner. It is also crucial to include a mechanism for ambient light rejection in the algorithm, or to choose a pulse oximetric sensor that performs such a function, such as with the sensor proposed in this work.

The presented algorithm is suitable as a drowning condition detector part of a whole safety system, which integrates the threat detection and transmission of the alarm signal to the lifeguards, or which reacts automatically by itself. Some systems of this type are proposed in [28,29,30,31].

## 8. Conclusions

The paper describes a decision algorithm that can be used in swimming pool safety monitoring systems. The algorithm allows for the triggering of alarms regarding potential danger in the case of detecting a persistent decrease in the oxygen saturation and heart rate of a subject. Reacting to a decrease in saturation value, instead of reacting to the saturation value threshold, significantly reduced the number of false-positive alarms. This article also presents methods for determining oxygen saturation and heart rate, as well as the nearest-peak selection method. The nearest-peak selection method is well-suited for eliminating motion artifacts.

## Figures and Tables

**Table 1:** Measured oxygen saturation variances.

PPG Frame nr	1	2	3	4	5	Average HR
HR (bpm)	74.27	73.65	73.71	71.83	72.05	73.1

**Table 2:** Heart rate variances.

	HR without MA Removal Variance	Mean HR with MA Removal Variance
Figure 14	13.51	2.76
Figure 15	13.62	1.84

**Table 3:** Measured oxygen saturation variances.

	Oxygen Saturation Variance
Finger	0.05
Wrist	3.01

**Table 4:** Measured oxygen saturation variances.

	HR Variance	SPO2 Variance
Tested algorithm	22.26	0.95
MUSIC	18.83	−
Wavelet transform	23.81	3.78
Decimation filter + periodogram	26.58	0.79

## Data Availability

Not applicable.
